# Evaluation of inhibition of F4ac positive *Escherichia coli* attachment with xanthine dehydrogenase, butyrophilin, lactadherin and fatty acid binding protein

**DOI:** 10.1186/s12917-015-0528-0

**Published:** 2015-09-15

**Authors:** Predrag Novakovic, Chandrashekhar Charavaryamath, Igor Moshynskyy, Betty Lockerbie, Radhey S. Kaushik, Matthew E. Loewen, Beverly A. Kidney, Chris Stuart, Elemir Simko

**Affiliations:** Western College of Veterinary Medicine, 52 Campus Drive, Saskatoon, SK S7N 5B4 Canada; Western University of Health Sciences, College of Veterinary Medicine, 309 East Second Street, Pomona, CA 91766 USA; Biology & Microbiology-Box 2140D, South Dakota State University, Brookings, SD 57007 USA

**Keywords:** F4 fimbriae, IPEC-J2 cells, *in vitro* adhesion, Milk fat globule membrane proteins

## Abstract

**Background:**

Neonatal and post-weaning colibacillosis caused by enterotoxigenic *E. coli* is responsible for substantial economic losses encountered by the pork industry. Intestinal colonization of young piglets by *E. coli* depends on the efficiency of bacterial attachment to host gastrointestinal epithelium that is mediated by fimbriae. We tested the effect of porcine individual milk fat globule membrane (MFGM) proteins on F4ac positive *E. coli* attachment to porcine enterocytes *in vitro*.

**Results:**

Butyrophilin, lactadherin and fatty acid binding protein inhibited fimbriae-dependent adherence of *E. coli* to enterocytes *in vitro*, while xanthine dehydrogenase did not. The inhibiting activity was dose-dependent for all three proteins, but the inhibiting efficiency was different.

**Conclusions:**

The results indicate that MFGM proteins may interfere with attachment of *E. coli* to porcine neonatal intestinal mucosa.

## Background

Post-weaning diarrhea (PWD) due to F4ac-positive enterotoxigenic *Escherichia coli* (ETEC) is an important cause of morbidity and mortality in weaned piglets [1]. Currently, PWD is controlled by various management strategies, use of antibiotics as feed supplements and/or immunization with vaccines containing fimbrial antigens. However, none of these control measures can completely eliminate PWD from modern swine production. In addition, continuous use of sub-therapeutic doses of antibiotics as feed supplements potentially leads to the emergence of genes encoding antimicrobial resistance in porcine microflora. These antimicrobial resistant genes may be incorporated by animal and human pathogens, potentially causing serious public health problems. Hence, there is a great demand to find alternative strategies for prevention and control of porcine post-weaning diarrhea (PWD). Many domestic animal species including pigs are born hypogammaglobulinemic and rely on sow’s milk for immune protection. Vaccination of sows efficiently protects piglets against ETEC infection only during the nursing period. However, after weaning, ingestion of antibodies and other potentially protective substances from sow’s milk is terminated, and piglets become susceptible to ETEC infection. Since the immune system of neonatal piglets is relatively naïve, the current vaccination strategies at that age are not sufficiently effective for protection against PWD [2]. Practices of sow vaccination contribute to prevention of ETEC infection in suckling piglets. Fimbriae-specific antibodies in sow milk, however, may decrease the efficacy of orally administered vaccine in neonatal piglets. In addition to ETEC-specific maternal immunoglobulins, porcine milk also contains a variety of non-immunoglobulin substances that can also interfere with ETEC attachment to enterocytes.

Atroshi et al., reported that porcine milk fat globule membrane (MFGM) can act as a target for binding of F4 positive *E. coli* [3]. Furthermore, it was demonstrated that porcine MFGM have the potential to inhibit binding of F4 fimbriae to porcine intestinal brush borders. Subsequently, we identified the individual proteins of porcine MFGM (i.e. lactadherin, butyrophilin, adipophilin, acyl-CoA synthetase and fatty acid binding protein) that have binding affinity for F4ac fimbriae of ETEC [4,5] and demonstrated that porcine lactadherin interfered with attachment of F4+ ETEC to intestinal *villi in vitro* [4]. However, inhibitory capacity of the rest of MFGM proteins against attachment of ETEC to enterocytes is not known. Therefore, the purpose of this study was to isolate the previously identified F4ac-binding proteins from porcine MFGM and to test their ability to inhibit attachment of F4ac-fimbriae or F4ac positive *E. coli* to primary porcine enterocytes or IPEC-J2 cell line using competitive enzyme-linked immunosorbent assay (ELISA).

## Methods

In this study we compared ability of enterotoxigenic *E. coli* to attach to cultured porcine enterocytes in the presence and in the absence of individually purified milk fat globule proteins. Biotinylated F4 positive *E. coli* was pre-incubated with each of purified proteins and allowed to attach to immobilized enterocytes in 96 wells microplate format. After washing off the excess of unattached bacteria, the plates were probed with streptavidin-conjugated reporter. The strength of the signal in each individual well was considered corresponding to efficiency of bacterial adherence.

### IPEC-J2 cell line and culture conditions

The IPEC-J2 [undifferentiated porcine intestinal epithelial cell line derived from porcine jejunum, a kind gift from Dr. Pradip Maitii (Nutratechglobal, Winnipeg, MB, Canada)] cells were seeded on cell culture flask (T75cm^2^, Corning, NY, USA) as described previously [6]. Briefly, IPEC-J2 cells were cultured and maintained in Dulbecco’s Modified Eagle Medium (DMEM)-Hank F12 (Gibco, Invitrogen Corporation, Grand Island, NY, USA) supplemented with 5 % fetal calf serum (FCS, Atlanta Biologicals, Lawrenceville, GA, USA), penicillin (100 IU/ml), streptomycin (100 μg/ml) (Invitrogen Corporation, Grand Island, NY, USA), and 5 ng/ml of epidermal growth factor (Sigma Chemical Co., St. Louis, MD, USA). IPEC-J2 cells were maintained in a humidified incubator in an atmosphere of 5 % CO_2_ and 95 % air at 37 °C. All experiments were carried out using cells passaged 12 to 60 times. After 4 to 5 days of culturing, the confluent cell monolayer was briefly rinsed with 1× PBS pH 7.4 (Gibco, Invitrogen Corporation, Grand Island, NY, USA) to remove all traces of serum. Five hundred microliters of 0.25 % Trypsin-EDTA solution (Gibco, Invitrogen Corporation, Grand Island, NY, USA) was added to the flask and incubated at 37 °C. Cells were observed under an inverted microscope and incubation was continued with trypsin-EDTA until the cell monolayer was completely dispersed (usually within 7 to 10 min). The detached cells were collected and centrifuged at 200 × *g* for 5 min, resuspended in ice cold phosphate-buffered saline (PBS) (1.6 mM NaH_2_PO_4_, 9.4 mM Na_2_HPO_4_, 154 mM NaCl, pH 7.4) to a density of 2 × 10^6^cells/ml before being used for the ELISA test. The continuous culture of the IPEC-J2 cells was maintained by seeding culture flasks at 1:4 or 1:5 ratios at each passage into T75cm^2^ flasks.

### Pig enterocyte isolation

The animal use protocol was reviewed and approved by the Animal Research Ethics Board (AREB) at the University of Saskatchewan and followed the principles established by the Canadian Council on Animal Care [7].

The isolation of fresh enterocytes was done by using a distended intestinal sac method as described previously [8]. Briefly, a six to eight day old piglet from Prairie Swine Center was euthanized with a halothane overdose (MTC Pharmaceuticals, Cambridge, ON, Canada) and subsequent exsanguination. An 80-cm segment of jejunum was dissected, and rinsed with a pre-incubation buffer [PBS with 0.2 mM phenylmethylsulfonyl fluoride (PMSF) and 0.5 mM dithiothreitol]. The caudal end was then clamped, and the segment was filled with isolation buffer (PBS with 1.5 mM EDTA, 0.2 mM PMSF, and 0.5 mM dithiothreitol) until fully distended. The cranial end was then clamped, and the distended jejunal segment was placed in pre-warmed saline in a 2 L beaker, kept on a shaking water bath at 37 °C. After 20 min of incubation [9], contents of the segment including isolated enterocytes were collected into a conical tube and centrifuged (400 × *g* for 3 min at 4 °C). The cell pellet obtained after centrifugation was resuspended in ice cold PBS and this washing step was repeated 4 times. Finally, the enterocytes were resuspended in ice cold PBS to a density of 2 × 10^6^ cells/ml before using in an ELISA test.

### F4ac-positive *Escherichia coli*

Enterotoxigenic F4ac-positive *Escherichia coli* (reference strain P97-2554B, O149:K91:F4ac^+^ EST1^+^LT^+^STa^+^STb^+^) isolated from the intestine of a pig with diarrhea at the faculty of Veterinary Medicine, University of Montreal, Saint-Hyacinthe, QC was kindly provided by Dr. Musangu Ngeleka, Prairie Diagnostic Services, Inc., Saskatoon, SK.

### Culture and use of F4ac-positive *Escherichia coli*

An overnight growth of bacterium in trypticase soy broth (TSB) was mixed in sterile glycerol to a final concentration of 15 % glycerol and stored at – 20 °C for future use. A loop full of glycerol stock culture was spread on a tryptic soy agar (TSA) plate with 5 % sheep blood and incubated at 37 °C overnight. Next day, a single colony was inoculated into 30 ml of TSB and incubated at 37 °C with 150 rpm shaking over night. The following day, the bacterial suspension was centrifuged (3000 × *g*, for 30 min at 25 °C) and the pellet was resuspended in carbonate buffer (100 mM NaCO_3,_ pH 8.2). Using a spectrophotometer (NanoDrop 2000C, Thermofisher Scientific, Wilmington, DE, USA), OD of the bacterial suspension was adjusted to 0.7 (A550 nm) against carbonate buffer as the blank value.

### Isolation and identification of F4ac fimbriae

F4ac fimbriae were isolated and purified according to a previously published method [10], the only modification being the introduction of filtration of solubilized F4ac fimbriae through 0.20 μm pore size filters (Nylon Membrane 0.2 μm 47 mm PK/100, Millipore Corporation, MA, USA) before each step of precipitation at pH 4 during the purification process to prevent aggregation [5]. The concentration of isolated fimbriae was determined with Quick Star Bradford protein assay (Bio-Rad Laboratories, Mississauga, Ont., Canada), the purity by Sodium Dodecyl Sulphate (SDS) Polyacrylamide Gel Electrophoresis (PAGE) separation and Coomassie staining and the identity by Western Blot technique using rabbit anti-F4ac polyclonal antibodies (Dr J.M. Fairbrother, Faculte de Medecine Veterinaire, Universite de Montreal, Saint-Hyacinthe, QB, Canada) as previously described [5]. The isolated F4ac fimbriae protein was stored at −20 °C in PBS.

### Isolation of xanthine dehydrogenase, butyrophilin, lactadherin and fatty acid binding protein

MFGM proteins were isolated from porcine milk fat according to previously published methods [11,12] with minor changes described previously [5].

Isolation of the four individual MFGM proteins of interest, namely, xanthine dehydrogenase (XDH), butyrophilin (BTN), lactadherin (LAD) and fatty acid binding protein (FABP) was done by electro-elution technique. Briefly, MFGM proteins were loaded in multiple lanes (100 μg/well) and separated in 12 % polyacrylamide gel by SDS PAGE using a large electrophoretic apparatus (Protean II xi Cell, Bio-Rad Laboratories, Mississauga, Ontario, Canada) under constant current of 24 mA for 5 h. After separation, the reference lane was cut off and the separated proteins were visualized by Coomassie staining as previously described [13]. The Coomassie-stained reference lane was lined up with the rest of the unstained gel to cut the regions of unstained proteins corresponding to individual separated and Coomassie-stained proteins in the reference lane. Unstained gel regions containing individual MFGM proteins were sliced in 1–2 cm segments and loaded into an electro-elution system (Elutrap, Whatman, Dassel, Germany). After 8 h of elution under 200 V in Tris-glycine buffer (25 mM Tris, pH 8.3; 192 mM glycine; 0.1 % Sodium Dodecyl Sulphate), eluted individual proteins were collected. The remaining SDS was removed from isolated proteins by dialysis (72 h, with changes of deionized water every 4 h, at 4 °C) using cellulose dialysis tubing with 8 kDa cut-off (Spectra/Por, Spectrum Laboratories, Inc., CA, USA). Finally, purity of the individual proteins eluted from the 12 % acrylamide gel was analyzed by Coomassie staining after SDS-PAGE separation, and the concentration was determined with Quick Star Bradford protein assay (Bio-Rad Laboratories, Mississauga, ON, Canada).

### Coating of microtiter plates with IPEC-J2 and pig enterocytes

Immobilization of IPEC-J2 cells and fresh pig enterocytes was done as previously published [14]. Briefly, 100 μ1 of 1 M lysine solution (in distilled water) was added to each well of 96-well, flat-bottomed polystyrene microtiter plates (Gibco BRL, Canadian Life Technologies, Burlington, ON, Canada) and incubated for at least 10 min (up to 2 h) at room temperature. After three washes with distilled water, 100 μ1 of a 1.25 % glutaraldehyde solution (in distilled water) was added to each well. The plates were incubated for exactly 5 min and then they were washed 2 times with distilled water to activate the plates. PBS (100 μ1) was immediately added to each well, followed by 50 μl of cell suspension (2× 10^6^ cells/ml, IPEC-J2 or freshly isolated pig enterocytes). The optimal numbers of cells for competitive ELISA were determined by checkerboard titration in direct ELISA, testing various numbers of cells with various numbers of biotinylated F4-positive *E. coli*. The cells were gently mixed and allowed to settle for 10 min at room temperature. The plates were then centrifuged for 10 min at 650 × *g*. The supernatant (100 μl) was aspirated with caution, so as not to disrupt the cell monolayer. The plates were then dried overnight at 37 °C and stored at room temperature until used (not longer than 10 days).

### Biotinylation of F4ac-positive *E. coli* and F4ac fimbriae

F4ac-positive *Escherichia coli* was cultured and a bacterial suspension with optical density of 0.7 at 550 nm in carbonate buffer was made as described above. 12.5 μ1 of reconstituted NHS-PEO_4_ Biotin in distilled water (EZ-Link NHS-PEO-Biotinylation kit, Pierce Biotechnology, Rockfor, IL, USA) was added to 1 ml of the bacterial suspension, mixed and incubated for 2 h at room temperature. The bacteria were then washed 3 times with PBS (3000 × *g*, 10 min at room temperature) and finally resuspended in PBS with 1 % (wt/vol) D-mannose (Sigma Chemical Co., St. Louis, MD, USA) to the original volume (5 ml) just before use in the ELISA test. The optimal numbers of bacteria (2 × 10^8^/ml of biotinylated F4ac-positive *E. coli*) for competitive ELISA were determined by checkerboard titration in direct ELISA, testing various numbers of biotinylated F4-positive *E. coli* with various numbers of porcine enterocytes.

Biotinylation of F4ac fimbriae was done according to the manufacturer instructions for biotinylation of proteins (EZ-Link NHS-PEO-Biotinylation kit, Pierce Biotechnology, Rockfor, IL, USA).

### Competitive ELISA for quantification of inhibition of adherence of F4ac-positive *E. coli*

The competitive ELISA test for quantification of inhibition of F4ac-positive *E. coli* adherence to IPEC-J2 cells with isolated MFGM proteins was done as previously published [14] with few modifications. The unoccupied plastic spaces in a 96-well microtiter plate containing immobilized IPEC-J2 was blocked by adding 300 μl/well of a 5 % solution of BSA in PBS. The plate was incubated for 3 h at 37 °C and washed 3 times in PBS (300 μl/well). Then, 1 ml of 2 × 10^8^ biotinylated F4ac-positive *E. coli* in 1 % PBS-D-mannose buffer had been pre-incubated with isolated xanthine dehydrogenase (5, 25 or 125 μg); butyrophilin (5, 25 or 125 μg); lactadherin (5, 25 or 125 μg), or fatty acid binding protein (5, 25 or 125 μg). Rabbit anti-F4ac polyclonal antibody was used as positive control (5, 25, or 125 μg of polyclonal serum proteins) (Dr J.M. Fairbrother, Faculte de Medecine Veterinaire, Universite de Montreal, Saint-Hyacinthe, QB, Canada), and rabbit anti-F6 polyclonal antibody (125 μg) (Dr J.M. Fairbrother, Faculte de Medecine Veterinaire, Universite de Montreal, Saint-Hyacinthe, QB, Canada) and lactalbumin (125 μg) (Gibco BRL, Canadian Life Technologies, Burlington, ON, Canada) as negative controls. In order to determine nonspecific binding, additional controls were also included, such as wells with only cells (immobilized IPEC-J2 or fresh enterocytes) without biotinylated F4ac-positive *E. coli,* and wells with PBS only without cells or biotinylated F4ac-positive *E. coli.* After 1 h of incubation of biotinylated F4ac-positive *E. coli* with test reagents at room temperature with gentle shaking, 50 μl was added to each well of the microtiter plate for ELISA. The plate was incubated for 40 min at 37 °C, and subsequently washed 3 times with PBS. Then 100 μl of PBS was added into each well and the plate was heat-fixed for 10 min at 60–65 °C. After that PBS was removed and horseradish peroxidase streptavidin conjugate (100 μl/well, 1:7000 dilution in PBS) (Vector Laboratories, Inc., Burlingame, CA, USA) was added to each well and incubated for 30 min at 37 °C. The plate was washed 3 times with PBS, and 100 μl of TMB microwell peroxidase substrate system (KPL, Gaithersburg, MD, USA) was added to each well. The color was allowed to develop in the dark for 15 to 30 min and then stop solution (2 N H_2_SO_4_, 100 μl/well) was added. ELISA optical density (OD) was measured at 450 nm within 30 min with Spectra Max 340 PC Spectrophotometer operated by SoftMax Pro software (Molecular Devices Corporation, Sunnyvale, CA, USA). The OD of test wells was plotted after deducting the average OD of wells treated with PBS only in order to account for non-specific background color development.

The competitive ELISA test for quantification of inhibition of F4ac-positive *E. coli* adherence to pig enterocytes with isolated MFGM proteins was done as described for IPEC-J2 cells, with the exception that freshly isolated enterocytes were used to coat microtiter plates.

### Competitive ELISA for quantification of inhibition of F4ac fimbriae attachment

The competitive ELISA for quantification of inhibition of F4ac fimbriae attachment to IPEC-J2 with MFGM proteins was performed similarly as described for F4ac-positive *E.coli*. This test was used to determine whether isolated MFGM protein mediated inhibition of F4ac-positive *E. coli* adherence to IPEC-J2 cells *in vitro* is because of the specific interaction between MFGM protein and F4ac fimbriae. The ability of biotinylated F4ac fimbriae to attach to IPEC-J2 cells and optimal concentration for competitive ELISA test were determined by direct ELISA, testing various concentrations of biotinylated F4ac fimbriae on IPEC-J2 coated microtiter plates. The concentration of 0.5 μg/ml of purified biotinylated F4ac fimbriae was determined as optimal and used in the competitive ELISA test as described for competitive ELISA for quantification of inhibition of adherence of F4ac positive *E. coli* to IPEC-J2 cells with MFGM proteins. Briefly, 0.5 μg/ml (molar concentration, 18.52 nM) of biotinylated F4ac fimbriae was incubated with 5 μg/ml (66.67 nM), 25 μg/ml (333.33 nM), or 125 μg/ml (1.67 μM) of xanthine dehydrogenase; 5 μg/ml (151.51 nM), 25 μg/ml (757.57 nM), or 125 μg/ml (3.79 μM) of butyrophilin; 5 μg/ml (212.77 nM), 25 μg/ml (1.06 μM), or 125 μg/ml (5.32 μM) of lactadherin; and 5 μg/ml (769.23 nM), 25 μg/ml (3.85 μM), or 125 μg/ml (19.23 μM) of fatty acid binding protein for 1 h at room temperature. The negative controls and other conditions of the test were the same as described above.

### Statistical analysis

The data were analyzed for statistical significance (*P* < 0.05) by ANOVA with Tukey’s multiple comparisons test (post-hoc test) using Prism 5 for Windows, version 5.04 (GraphPad Software, Inc., La Jolla, CA, USA). The results were expressed in the form of the optical density generated by biotinylation of bacteria and fimbria. ANOVA test was used for analysis of normally distributed data of quadruplicates (four wells per sample point) in each independent experiment. Therefore, data were analysed as parametric and each column of Figs. 3, 4 and 5 represents quadruplicate of measurements in a separate representative experiment. Each experiment was repeated at least three times to ensure its reproducibility but the data were not combined, therefore the adjustment for multiple testing was not necessary. Statistical significance was determined for the differences between groups of replicates within each independent experiment.

## Results

Butyrophilin, lactadherin and fatty acid binding protein inhibited fimbriae-dependent adherence of *E. coli* to enterocytes *in vitro*, while xanthine dehydrogenase did not. The inhibiting activity was dose-dependent for all three proteins, but the inhibiting efficiency was different.

### Adherence of F4ac-positive *E. coli* to IPEC-J2 cell and pig enterocytes

In order to assess the ability of each MFGM protein of interest to inhibit F4ac-positive *E. coli* adherence to porcine intestine, we employed an already established cellular system (IPEC-J2 cell line) [6] and confirmed that F4ac-positive *E. coli* adheres to both IPEC-J2 cells and pig enterocytes in a slide adherence assay (data not shown).

### Isolation of F4ac fimbriae from F4ac-positive *E. coli*

**Fig. 1:**
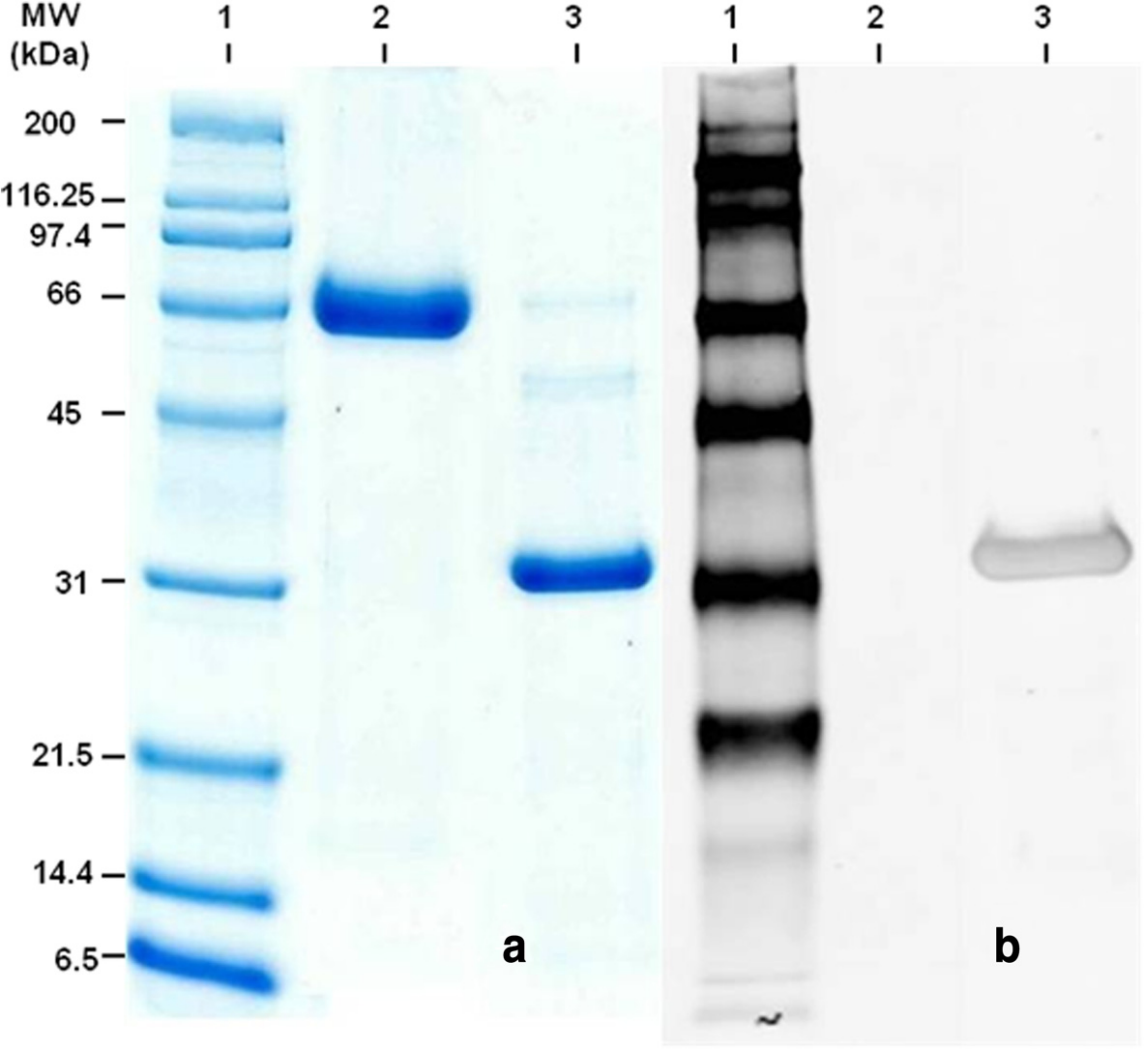
Isolation of F4ac fimbriae from F4ac-positive *E. coli*. F4ac fimbriae isolated from F4ac-positive *E. coli*, subjected to 12 % SDS-PAGE, and visualized by Coomassie Blue staining (a) and detected by Western blotting using primary rabbit anti-F4-polyclonal antibodies and secondary anti-rabbit Cy5 antibodies (b). Lane 1 – (a) Molecular weight markers; (b) ECL Flex Rainbow Molecular Weight Markers; Lane 2 - Bovine serum albumin as negative control; Lane 3- isolated F4ac fimbriae

We isolated F4ac fimbriae from bacterial culture, analyzed its purity by SDS-PAGE separation and Coomassie staining and confirmed its identity by Western blot (Fig. 1).

### Isolation of MFGM proteins

**Fig. 2:**
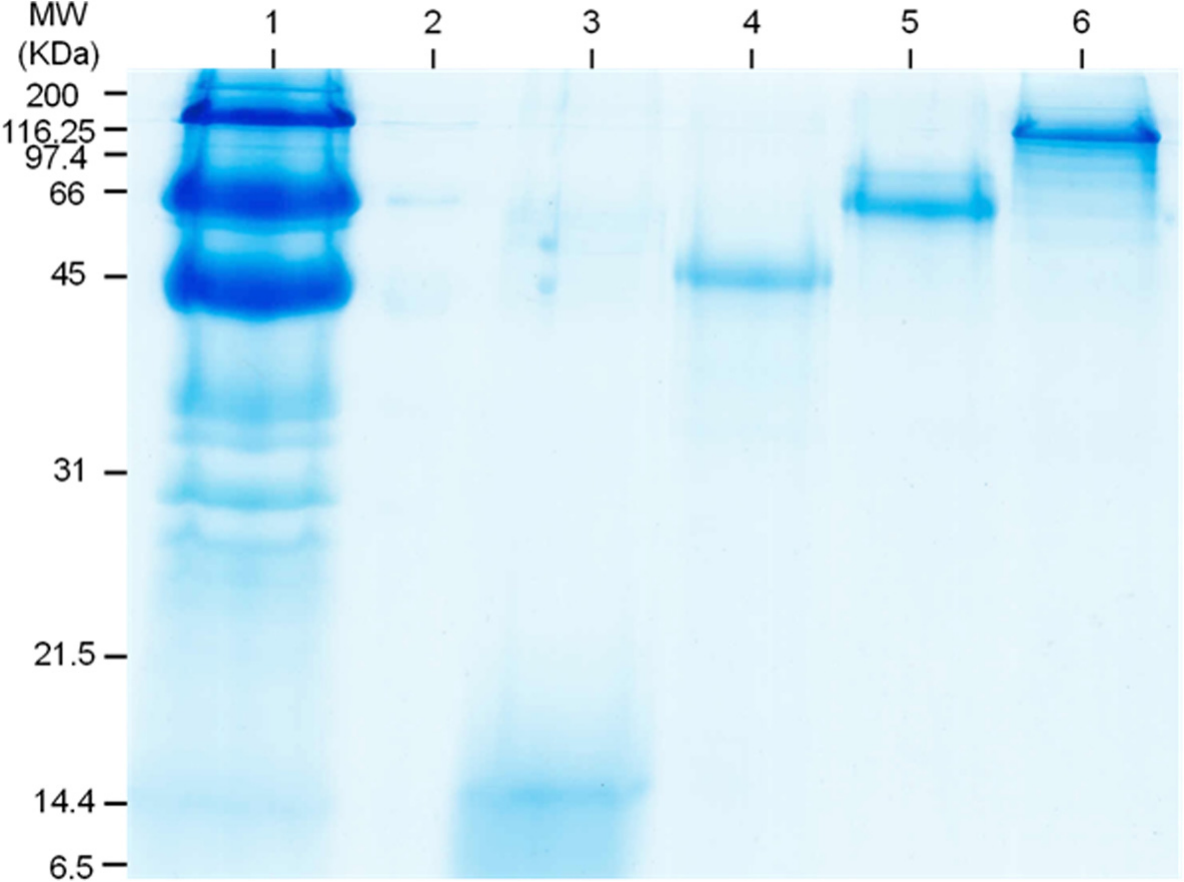
Isolation of MFGM proteins. Milk Fat Globule Membrane (MFGM) proteins isolated from porcine milk, subjected to 12 % SDS-PAGE separation, and visualized by Coomassie Blue staining (Lane 1 – MGFM proteins; Lane 2 – empty lane; Lane 3 – Electroeluted fatty acid binding protein; Lane 4 – Electroeluted lactadherin; Lane 5 – Electroeluted butyrophilin; Lane 6 – Electroeluted xanthine dehydrogenase)

We isolated xanthine dehydrogenase, butyrophilin, lactadherin, and fatty acid binding protein by electro-elution. To confirm the purity of individual proteins we separated those by SDS-PAGE, and visualized by Coomassie blue stain (Fig. 2). Identities of milk fat globule membrane proteins were confirmed using mass spectroscopy. The identification and ID data have been published elsewhere [5].

### Quantification of inhibitory effect of MFGM proteins on attachment of F4ac-positive *E. coli* to the IPEC-J2 cells

**Fig. 3:**
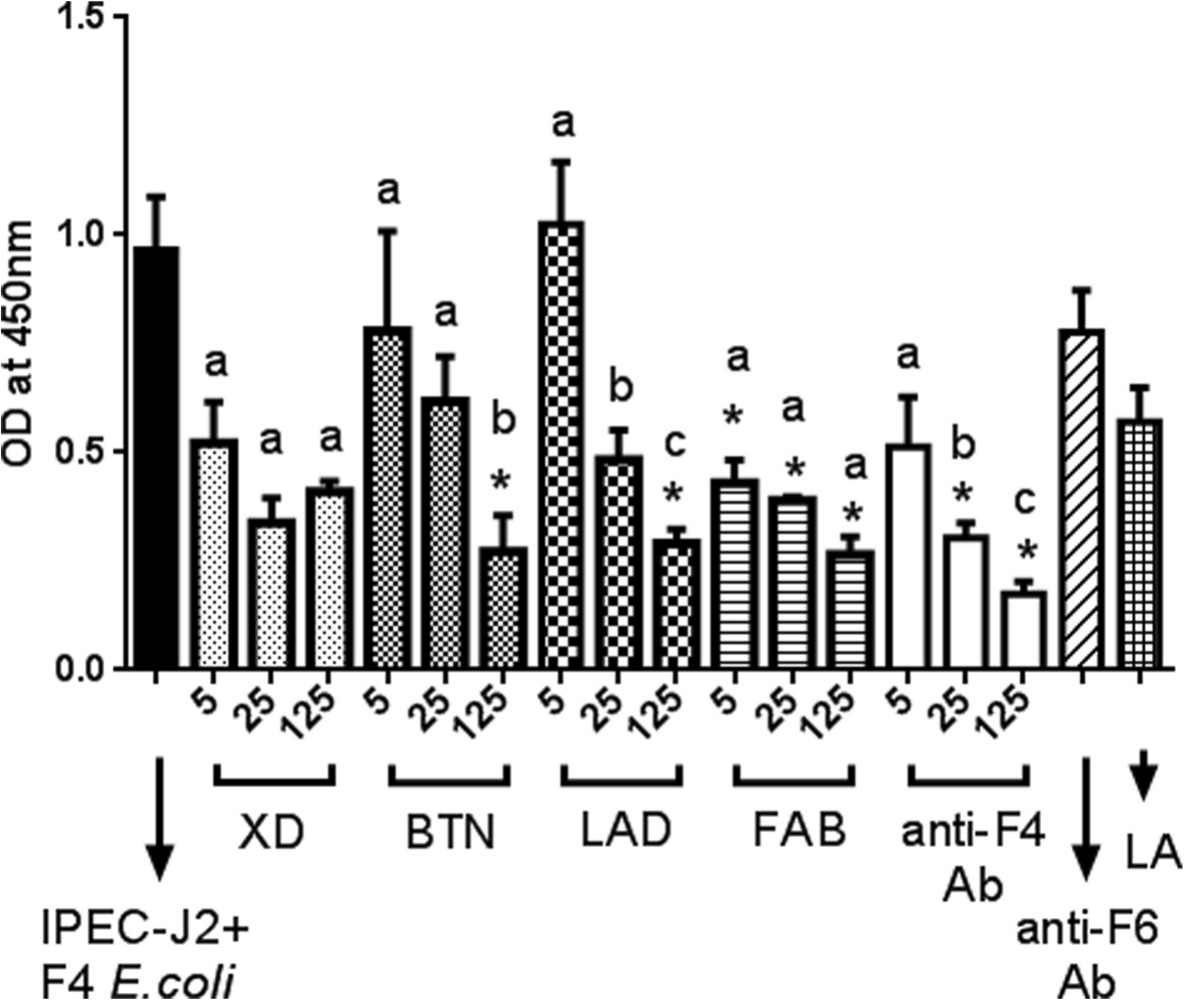
Quantification of inhibitory effect of MFGM proteins against attachment of F4ac-positive *E. coli* to the IPEC-J2 cells. Inhibition of F4ac-positive *E. coli* attachment to IPEC-J2 cells with various concentrations (5, 25 or 125 μg/ml) of xanthine dehydrogenase (XD), butyrophilin (BTN), lactadherin (LAD), fatty acid binding protein (FAB); positive (rabbit anti-F4ac polyclonal antibody) and negative (rabbit anti-F6 polyclonal antibody or Lactalbumin) controls. Asterisk (*) indicates statistically significant difference between an individual dilution and all of the negative controls (untreated JPEC-J2 + F4ac-positive *E. coli*, Lactalbumin or anti-F6 antibodies), (*P* < 0.05). Different letters (a), (b) and (c) indicate statistically significant differences (*P* < 0.05) between individual dilutions of the same milk fat globule membrane protein. Error bar indicates Standard Deviation. Each column represents mean of quadruplicate measurements in a single representative experiment

We performed different experiments to analyze the inhibitory effect of MFGM proteins against F4ac-positive *E. coli* adherence by competitive ELISA. Competitive ELISA test for quantification of inhibition of adherence of F4ac-positive *E. coli* to IPEC-J2 cells with various concentrations (5, 25 or 125 μg) of MFGM proteins in comparison to positive (rabbit anti-F4ac polyclonal antibody) and negative controls (rabbit anti-F6 polyclonal antibody and lactalbumin) demonstrated that lactadherin, butyrophilin, and fatty acid binding protein inhibit adherence of F4ac-positive *E. coli* to the IPEC-J2 cells in a dose dependent manner (Fig. 3).

### Quantification of inhibitory effect of MFGM proteins on attachment of F4ac-positive *E. coli* to the pig enterocytes

**Fig. 4:**
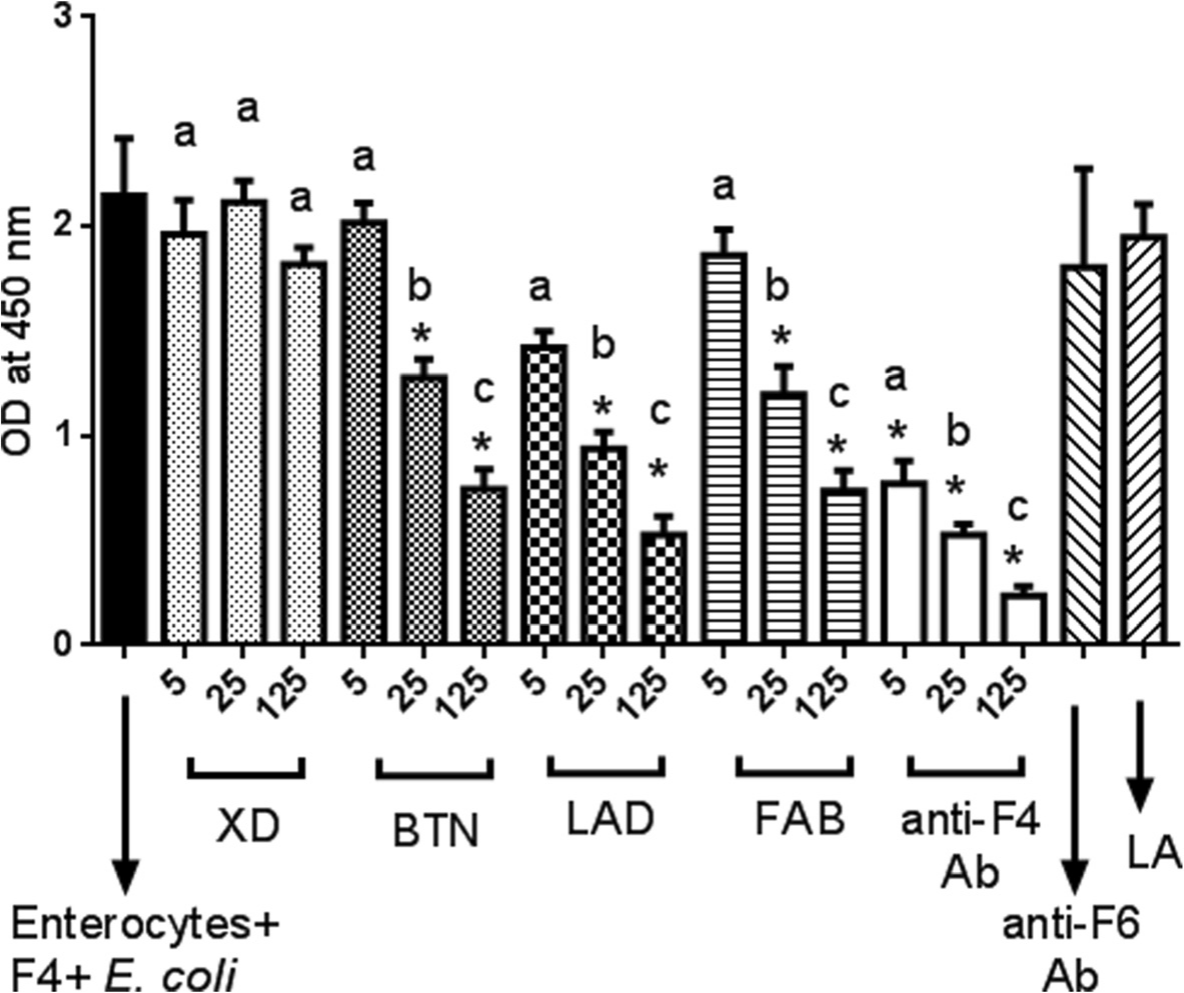
Quantification of inhibitory effect of MFGM proteins against attachment of F4ac-positive *E. coli* to the pig enterocytes. Inhibition of F4ac-positive *E. coli* attachment to porcine enterocytes with various concentrations (5, 25 or 125 μg/ml) of xanthine dehydrogenase (XD), butyrophilin (BTN), lactadherin (LAD), fatty acid binding protein (FAB); positive (rabbit anti-F4ac polyclonal antibody) and negative (rabbit anti-F6 polyclonal antibody or Lactalbumin) controls. Asterisk (*) indicates statistically significant difference between an individual dilution and all of the negative controls (untreated enterocytes + F4ac-positive *E. coli*, Lactalbumin or anti-F6 antibodies), (*P* < 0.05). Different letters (a), (b) and (c) indicate statistically significant differences (*P* < 0.05) between individual dilutions of the same milk fat globule membrane protein. Error bar indicates Standard Deviation. Each column represents mean of quadruplicate measurements in a single representative experiment

We tested the biological relevance of our *in vitro* system IPEC-J2 by repeating Competitive ELISA test with freshly isolated pig enterocytes and obtained very similar results (Fig. 4). Namely, lactadherin, butyrophilin, and fatty acid binding protein inhibited adherence of F4ac-positive *E. coli* to enterocytes, while xanthine dehydrogenase did not.

### Quantification of inhibitory effect of MFGM proteins on attachment of F4ac fimbriae to the IPEC-J2 cells

**Fig. 5:**
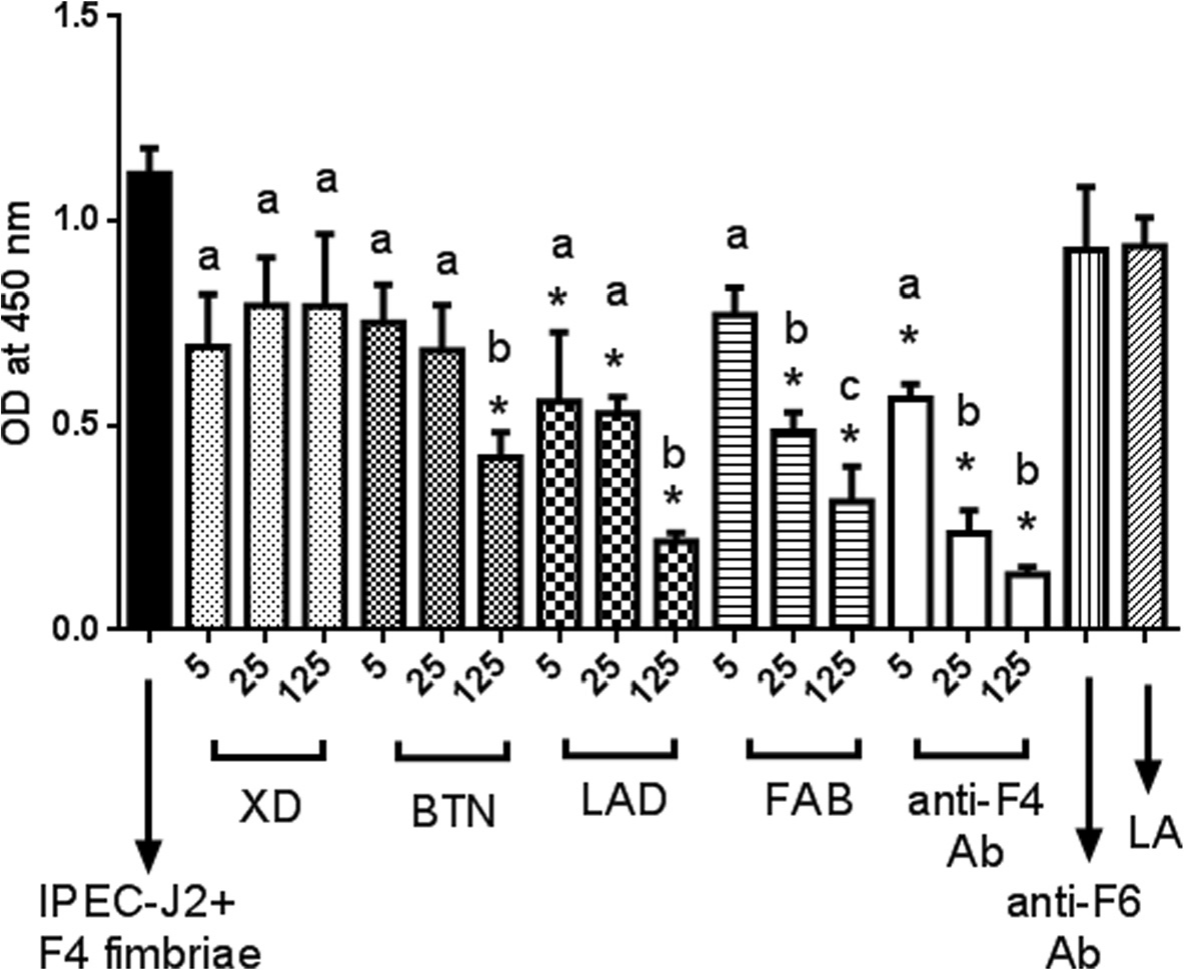
Quantification of inhibitory effect of MFGM proteins against attachment of F4ac fimbriae to the IPEC-J2 cells. Inhibition of F4ac fimbriae attachment to IPEC-J2 cells with various concentrations (5, 25 or 125 μg/ml) of xanthine dehydrogenase (XD), butyrophilin (BTN), lactadherin (LAD), fatty acid binding protein (FAB); positive (rabbit anti-F4ac polyclonal antibody) and negative (rabbit anti-F6 polyclonal antibody or Lactalbumin) controls. Asterisk (*) indicates statistically significant difference between an individual dilution and all of the negative controls (untreated JPEC-J2 + F4ac, Lactalbumin or anti-F6 antibodies), (*P* < 0.05). Different letters (a), (b) and (c) indicate statistically significant differences (*P* < 0.05) between individual dilutions of the same milk fat globule membrane protein. Error bar indicates Standard Deviation. Each column represents mean of quadruplicate measurements in a single representative experiment

In order to confirm that inhibition of F4ac-positive *E. coli* adherence to IPEC-J2 cell line *in vitro* is because of the interaction between MFGM protein and F4ac fimbriae, we designed the Competitive ELISA test for quantification of inhibition of F4ac fimbriae attachment to IPEC-J2 cells and confirmed that inhibition of attachment is most likely mediated by interaction between individual MFGM proteins and F4ac fimbriae (Fig. 5). Based on comparative inhibitory molar concentrations of MFGM proteins and F4ac, it appeared that 5 molecules of LAD, 50 molecules of BTN, and 200 molecules of FABP were needed to inhibit attachment of one molecule of F4ac fimbriae to IPEC-J2 cells.

## Discussion

In the current study, we demonstrated that butyrophilin, lactadherin and fatty acid binding protein interfere with attachment of F4ac-positive *E. coli* to the IPEC-J2 cells or porcine enterocytes. The inhibitory effect exhibited by these MFGM proteins appears to be in a dose-dependent manner and due to an interaction with F4ac fimbriae.

It has long been known that porcine milk fat globule surrounded by milk fat globule membrane (MFGM) binds to F4-positive *E. coli* [3]. We demonstrated previously by two independent techniques (overlay Western blot and affinity chromatography) that acyl-CoA synthetase 3, butyrophilin, adipophilin, lactadherin, and fatty acid binding protein 3 have binding affinity for F4ac fimbriae *in vitro* [5]. In this study we confirmed not only that they interact with F4ac fimbriae, but they also exhibit inhibitory effects against binding of F4ac-positive *E. coli* to enterocytes and non-transformed porcine jejunal epithelial cell line (IPEC-J2) *in vitro*.

Lactadherin is a 46 kDa peripheral glycoprotein, and a major protein on MFGM. Lactadherin has an N-terminal epidermal growth factor (EGF)-like domain, which mediates binding to α_v_β_5_ integrin [15]. Multiple functions of lactadherin have been reported so far. Lactadherin is involved in cellular adhesion, neovascularization and clearance of apoptotic cells [16–18]. Human lactadherin is associated with protection of human infants against rotaviral infection [19]. Porcine lactadherin was reported to have high affinity for F4ac fimbriae [4,5] and able to prevent binding of F4ac positive *E. coli* to intestinal villi *ex vivo* [4]. Consistent with the previous report [4], lactadherin was found in this study to inhibit the attachment of F4ac positive *E. coli* to primary enterocytes and a jejunal cell line and attachment of purified F4ac fimbriae to a jejunal cell line. Compared to butyrophilin and fatty acid binding protein, lactadherin appears to be the most potent inhibitor of *E. coli* attachment to enterocytes. Based on comparative inhibitory molar concentrations of lactadherin and F4ac, it seems that a minimum of 5 molecules of lactadherin is needed to inhibit binding of 1 molecule of F4ac fimbria to a jejunal cell line.

Butyrophilin is the most abundant protein of MFGM [20]. It is a type I membrane glycoprotein and belongs to the immunoglobulin superfamily [21]. Milk fat secretion is mediated by a complex of butyrophilin, xanthine dehydrogenase and adipophilin [17]. The biological importance of butyrophilin has not been defined so far. We previously reported that it has a binding affinity to F4ac fimbriae *in vitro*, and here we confirmed that it inhibits F4ac positive *E. coli* attachment to enterocytes by interaction with F4ac fimbriae.

Fatty acid binding protein of MFGM is a 13 kDa protein [22]. Initially it was known as mammary-derived growth inhibitor, because it inhibited growth of mammary carcinoma cells [23]. Fatty acid binding protein in MFGM consists of a mixture of heart type and adipocyte type fatty acid binding protein [24]. Bovine mammary fatty acid binding protein does not appear to be glycosylated. The function of milk fatty acid binding protein is not yet clear [22]. Previous studies demonstrated that fatty acid binding protein from porcine milk interacts with F4ac fimbriae of ETEC *in vitro* [4,5]. In this study we demonstrated that fatty acid binding protein inhibits F4ac positive *E. coli* attachment to enterocytes and IPEC-J2 cell line.

Xanthine dehydrogenase is present in many mammalian tissues, but it is a major constituent of the MFGM. Xanthine dehydrogenase is a complex enzyme comprised of two identical 147 kDa subunits [25], which catalyzes the oxidation of purines to uric acid by addition of oxygen from H_2_O [22]. Xanthine dehydrogenase has been suspected to play an important role during the secretion of milk fat, facilitated by formation of a complex with butyrophilin and adipophilin [26]. Reactive oxygen species generated by xanthine dehydrogenase may also act as antibacterial components. In our previous study, xanthine dehydrogenase was isolated by F4ac-affinity chromatography together with other F4ac-fimbrial binding proteins, but its interaction with F4ac fimbriae was not detected with overlay Western blot [5] accordingly, we suspected that the identification by affinity chromatography of xanthine dehydrogenase as a F4ac-binding protein was a false positive result. This was further confirmed in the current study by evidence that xanthine dehydrogenase did not inhibit attachment of F4ac positive *E. coli* to enterocytes or IPEC-J2 cell line nor purified F4ac fimbriae to IPEC-J2 cell line.

The inhibition of attachment of 0.5 μg/ml of F4ac fimbriae to IPEC-J2 cells with various concentrations of candidate MFGM proteins was tested in Competitive ELISA. Results suggested that only 5, 25 or 125 μg/ml of lactadherin and 25 or 125 μg/ml of butyrophilin and fatty acid binding protein were significant competitors. Knowing the protein concentration used in an assay and calculated molecular weight, we calculated the molarity of each protein sufficient to inhibit fimbrial binding to IPEC-J2 cells. When molar concentrations of MFGM proteins were compared with F4ac fimbriae molarity, it appeared that 5 molecules of lactadherin, 50 molecules of butyrophilin, and 200 molecules of fatty acid binding protein were needed to inhibit attachment of one molecule of F4ac fimbria to IPEC-J2 cells.

Enterotoxigenic strain of *E. coli*, isolated from a clinical case of porcine diarrhea, was used for this study. ETEC is a cause of neonatal and post-weaning diarrhea in pigs. The mechanism by which MFGM proteins inhibit attachement of ETEC to epithelial cells is not known but we suspect that glycan moieties of these glycoprotein interact with bacterial fimbriae. Identification and subsequent addition of these carbohydrates to porcine feed could potentially be used as non-antibiotic feed additives for prevention of porcine postweaning diarrhea. We are currently characterizing glycan portion of some of these proteins.

## Conclusion

In conclusion, lactadherin, butyrophilin and fatty acid binding protein reduce attachment of F4ac-positive *E. coli* to primary enterocytes and jejunal cell line (IPEC-J2) *in vitro*. The mechanism of this inhibition was not determined, but previous studies on lactadherin demonstrated that the glycosylated portion of lactadherin played an important inhibitory role and served as receptor analogues for F4ac fimbriae [4]. Future studies will be needed to determine the inhibitory importance of glycan portions of butyrophilin and fatty acid binding protein, and the potential importance of MFGM proteins in prevention of enteric colibacillosis *in vivo*.

## Abbreviations

PWD: Post-weaning diarrhea
ETEC: Enterotoxigenic *Escherichia coli*
MFGM: Milk fat globule membrane
DMEM: Dulbecco’s Modified Eagle Medium
FCS: Fetal calf serum
PBS: Phosphate-buffered saline
ELISA: Enzyme-linked immunosorbent assay
AREB: Animal Research Ethics Board
CCAC: Canadian Council on Animal Care
TSB: Trypticase soy broth
PAGE: Polyacrylamide gel electrophoresis
XDH: Xanthine dehydrogenase
BTN: Butyrophilin
LAD: Lactadherin
FABP: Fatty acid binding protein
kDa: Kilodalton
OD: Optical density
WCVM: Western College of Veterinary Medicine
